# Optimizing Maize Production and Soil Microbiome Structure Through Reduced Chemical Nitrogen Supplemented with Organic Fertilizer

**DOI:** 10.3390/plants14152275

**Published:** 2025-07-24

**Authors:** Jian Zhang, Yaoyao Li, Jiawei Yuan, Lu Wang, Guoying Wei, Zhejun Liang

**Affiliations:** Cotton Research Institute, Shanxi Agricultural University, Yuncheng 044000, China; liyaoyao01@163.com (Y.L.); yjwsxnky@126.com (J.Y.); wwyouc@126.com (L.W.); weiguoying2025@163.com (G.W.)

**Keywords:** maize, integrated application of chemical and organic fertilizers, soil microorganisms, enzymes, grain yield

## Abstract

This study investigated the effects of reduced nitrogen combined with an organic fertilizer on maize yield, soil microbial communities, and enzyme activities to optimize fertilization strategies. A field experiment on cinnamon soil in Yuncheng, Shanxi, was conducted and included six treatments: no fertilizer (CK), conventional N (NC0, 180 kg N/ha), sole organic fertilizer (CN0, 3000 kg/ha), and reduced-N + organic fertilizer (CN1: 90 kg N/ha + 3000 kg/ha; CN2: 135 kg N/ha + 3000 kg/ha; and CN3: 180 kg N/ha + 3000 kg/ha). We analyzed yield components, soil nutrients, urease and invertase activities, and bacterial community structure (16S rRNA sequencing). The key results are as follows: CN1 achieved the highest yield (9764.87 kg/ha), which was 46.8% higher than CK. CN2 maintained comparable yields while delivering higher enzyme activities and microbial abundance, positioning this strategy as suitable for soil remediation. Co-application enriched two beneficial phyla, Proteobacteria and Planctomycetota (19% in CN2), with Proteobacteria positively correlating with urease activity and alkali-hydrolyzable N (*p* < 0.05), while Verrucomicrobiota negatively correlated with urease activity. In conclusion, 25–50% N reduction with an organic fertilizer (3000 kg/ha) synergistically enhances yield, soil enzymes, and beneficial microbiota, supporting sustainable high-yield agriculture with improved soil fertility.

## 1. Introduction

In the production of maize (*Zea mays* L.), one of the world’s three major food crops, the issue of irrational fertilizer use is becoming increasingly prominent [1]. Over the last century, the widespread application of chemical fertilizers has significantly boosted maize yields [2]. Among these, nitrogen fertilizer application promotes canopy development, increases the leaf area index, enhances the accumulation of photosynthetic products, and promotes the transfer of these products to the grains [3], making it a crucial means of enhancing and stabilizing yields in maize production. However, the excessive application of chemical fertilizers not only leads to the waste of agricultural resources but also results in crop yield reduction, environmental pollution, and the occurrence of soil secondary salinization [4]. Long-term fertilizer application can cause soil compaction, acidification, and a decline in fundamental soil fertility [5]. Therefore, the precise regulation of chemical fertilizer application is of significant importance for improving fertilizer use efficiency, protecting soil ecology and fertility, and reducing production costs.

The combined application of chemical fertilizers and organic fertilizers is an important strategy for improving soil fertility and increasing crop yields [6]. The application of organic fertilizers can not only improve the soil environment and enhance soil nutrient content [7], but can also increase crop nitrogen accumulation and reduce environmental pollution [8,9]. However, the nutrients in organic fertilizers require microbial decomposition in the soil before becoming available for plant uptake and utilization, meaning they cannot provide sufficient nutrition during the early growth stages of crops. The combined application of chemical and organic fertilizers resolves this contradiction through temporal nutrient complementarity: chemical fertilizers provide readily available nutrients during crop germination and seedling stages, while organic fertilizers continuously release nutrients during the middle and late growth stages, establishing a “fast-acting + slow-release” nutrient supply mode. This combined application strategy reduces chemical fertilizer usage while achieving stable and increased crop yields through enhanced soil fertility, thus becoming a vital technical pathway for sustainable agricultural development [10]. Long-term fixed-site research indicates that organic fertilizer application exerts a long-term regulatory effect on maize yield formation. Increasing organic fertilizer application can partially replace chemical fertilizers to ensure crops maintain high-yield levels [11]. The long-term combined application of organic and inorganic fertilizers can enhance soil fertility and promote the formation of a fertile plow layer while safeguarding yields [12]. Research on optimal application ratios indicates that substituting 50% of synthetic fertilizer with organic amendments represents a balanced approach for maintaining crop yield, soil fertility, and economic returns [13,14]. Long-term studies demonstrate that manure-based nitrogen substitution confers sustained benefits for maize production, including enhanced nitrogen uptake, reduced ammonia emissions, and improved soil carbon storage [15]. The high productivity under organic fertilization is sustained through synergistic plant–microbial interactions in the rhizosphere [16,17]. Notably, integrated organic–mineral fertilization produces the highest maize yield increases compared to the sole application of either fertilizer type [18,19,20].

Microorganisms are pivotal agents in soil material transformation and energy flow. Rhizosphere microorganisms enhance the effective utilization of soil nutrients by releasing enzymes to decompose organic matter and participating in the transformation and cycling of elements, such as carbon, nitrogen, and phosphorus. They serve as a key driving force for agricultural production [21]. Consequently, the structure of the soil microbial community and enzyme activity are vital indicators for evaluating soil fertility [22,23]. During their growth and reproduction, the mycelia and cell walls of soil microorganisms bind soil particles, contributing to the formation and stabilization of soil aggregates [24] and thus improving the soil microenvironment [25]. The exogenous application of organic fertilizers is a key method for enhancing soil organic matter (SOM). The decomposition of organic fertilizers increases the diversity and abundance of carbon sources in the soil, providing more sufficient nutrients for microbial growth and reproduction, thereby enhancing microbial community stability. Since microbial activity directly influences soil nutrient cycling, this practice may improve soil fertility over multiple growing seasons [26]. Previous research has demonstrated that the combined application of organic and chemical fertilizers can improve the soil microenvironment and nutrient supply, increase soil microbial abundance and diversity, and optimize the soil microbial community structure, thereby synergistically enhancing both inherent soil fertility and crop yield [27,28,29]. The combined application of organic and chemical fertilizers increases the complexity of the soil microbial network and enhances soil multifunctionality. Sordariomycetes was identified as a key taxon driving this improvement in soil multifunctionality [30]. Research revealed that the long-term application of an organic fertilizer effectively increases the relative abundance of Proteobacteria and Bacteroidetes in the peanut rhizosphere, while the diversity of rhizosphere bacteria showed a significant positive correlation with peanut yield [31]. Studies indicated that the complexity and stability of the bacterial community structure correspond to increases in aboveground crop biomass [32]. Experiments involving the combined application of different types of organic materials with chemical fertilizers investigated the relationships between soil nutrients, enzyme activity, and microorganisms [33]. The study concluded that combined application effectively enhances soil extracellular enzyme activity and increases soil bacterial abundance, thereby altering its community structure. This promotes the cycling of soil materials and energy, improving the soil environment. The interaction mechanisms between crops and soil microorganisms constitute a key interdisciplinary research focus in agronomy and ecology. Investigating these mechanisms is essential for elucidating how microorganisms regulate productivity and for advancing the application of microbiology in developing high-yielding, high-quality, and sustainable modern agriculture.

However, current research focusing on soil improvement and enhanced crop production efficiency through the application of organic fertilizers or amendments has paid relatively little attention to the interplay between crop yield, soil nutrients, and soil microorganisms. This study addresses this gap by analyzing crop yield, soil nutrient status, enzyme activity, and the structure of the maize rhizosphere bacterial community under different application rates. The study aims to select the optimal combined application rate of organic and chemical fertilizers and investigate the interaction mechanisms between crops, soil, and microorganisms, thereby providing a theoretical basis for enhancing both maize yield and inherent soil fertility in maize production systems.

## 2. Results

### 2.1. Maize Yield and Yield Components Under Reduced Nitrogen Combined with Organic Fertilizer Application

As shown in Table 1, maize yield was significantly increased in treatments receiving the chemical fertilizer (NC0, CN1, CN2, CN3) compared to both the no-fertilizer control (CK) and the treatment receiving only the organic fertilizer (CN0). CK exhibited the lowest 1000-kernel weight. Fertilization treatments significantly increased 1000-kernel weight by 18.64% to 30.10% compared to CK. CK also showed the lowest number of grains per row, while fertilization treatments significantly increased grains per row by 7.21% to 15.81% relative to CK. CN3 produced the highest number of ears per hectare, being 18.18% higher than CN0 (which had the lowest ear count), with a significant difference between these two treatments. However, no significant differences in rows per ear were observed among any treatments.

### 2.2. Soil Nutrient Content and Enzyme Activity Under Reduced Nitrogen Combined with Organic Fertilizer Application

As presented in Table 2, the application of both the nitrogen fertilizer and organic fertilizer increased soil organic matter (SOM) and alkali-hydrolyzable nitrogen (AN) content. Compared to the CK treatment, the NC0 and CN0 treatments showed increases in SOM by 6.73% and 23.29% and in AN by 9.67% and 13.37%, respectively. Notably, the application of the organic fertilizer resulted in more substantial increases. The application of reduced chemical nitrogen combined with organic fertilizer showed an increasing trend in soil nutrient content and enzyme activity, though the differences were not statistically significant. Specifically, the CN1 treatment exhibited the largest increase in AN (35.30%), whereas the CN2 treatment showed the largest increases in SOM (27.30%) and urease activity (13.80%). The urease activity was lowest in the NC0 treatment (3752.9 μg NH^4+^-N g^−1^ d^−1^). Urease activity significantly increased with organic fertilizer application, reaching its highest value of 4878.6 μg NH^4+^-N g^−1^ d^−1^ in the CN2 treatment, 23.07% higher than that in NC0. Invertase activity was highest in the treatment receiving only organic fertilizer (CN0), significantly exceeding the levels in both CK and NC0. Soil pH was higher in treatments receiving organic fertilizer (CN0, CN1, CN2, and CN3) compared to CK and NC0. Among the reduced nitrogen treatments (CN1, CN2, and CN3), soil pH showed a decreasing trend with decreasing nitrogen reduction (i.e., increasing N input).

### 2.3. Soil Microbial Community Alpha Diversity Under Reduced Nitrogen Combined with Organic Fertilizer Application

**Figure 1:**
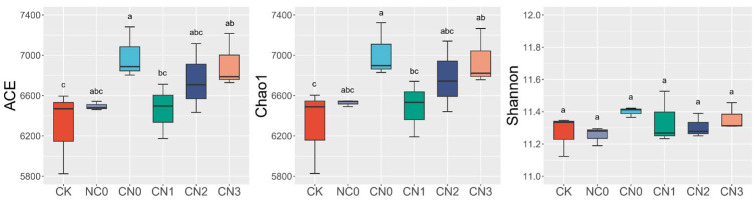
Box plots of bacterial community alpha diversity indices in maize rhizosphere soil under different treatments. Different lowercase letters indicate significant differences among treatments at *p* < 0.05. NC0: 180 kg N/ha; CN0: 3000 kg OF/ha; CN1: OF (3000 kg/ha) + Reduced N (90 kg N/ha, 50% reduction); CN2: OF (3000 kg/ha) + Reduced N (135 kg N/ha, 35% reduction); CN3: OF (3000 kg/ha) + Conventional N (180 kg N/ha, 0% reduction).

An alpha diversity analysis was conducted on the bacterial communities in the maize rhizosphere soil across different treatments (Figure 1). The treatment with conventional organic fertilizer application (CN0) exhibited the highest Chao1 index and Shannon index. In contrast, the lowest Chao1 index and Shannon index were observed in the no-fertilizer control (CK) and conventional nitrogen fertilization treatment (NC0), respectively. However, no significant differences were observed in the Shannon indices among all treatments, suggesting that the fertilization regime had limited impact on the overall evenness of the soil bacterial community. As illustrated in Figure 1, within the treatments combining organic and chemical fertilizers (CN0, CN1, CN2, and CN3), the indices of bacterial community richness (Chao1) and evenness (Shannon) showed an increasing trend with the increasing nitrogen application rate.

### 2.4. Soil Microbial Community Structure Under Reduced Nitrogen Combined with Organic Fertilizer Application

**Figure 2:**
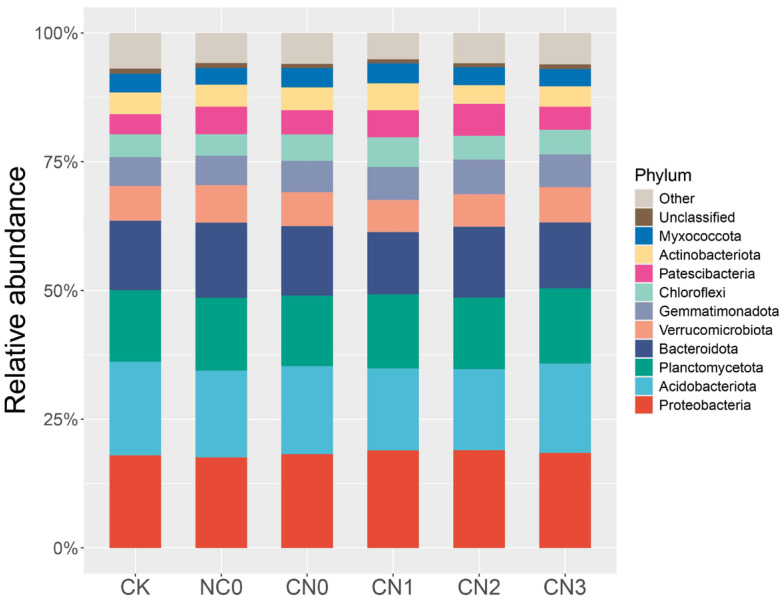
Relative abundance of dominant bacterial phyla in maize rhizosphere soil across treatments. NC0: 180 kg N/ha; CN0: 3000 kg OF/ha; CN1: OF (3000 kg/ha) + Reduced N (90 kg N/ha, 50% reduction); CN2: OF (3000 kg/ha) + Reduced N (135 kg N/ha, 35% reduction); CN3: OF (3000 kg/ha) + Conventional N (180 kg N/ha, 0% reduction).

Based on the annotation of sequencing results for each treatment, the phyla composition information of the microbial communities in each sample was obtained and presented as the relative abundance. As shown in Figure 2, the top ten bacterial phyla in terms of relative abundance across all treatments were Proteobacteria, Acidobacteriota, Planctomycetota, Bacteroidota, Verrucomicrobiota, Gemmatimonadota, Chloroflexi, Patescibacteria, Actinobacteriota, and Myxococcota. Among these, the dominant phyla with relative abundances exceeding 10% were Proteobacteria, Acidobacteriota, Planctomycetota, and Bacteroidota. In treatment CN2, Proteobacteria exhibited the highest relative abundance (19%), representing increases of 1.02% and 1.41% compared to CK and NC0, respectively. Bacteroidota also reached its highest relative abundance (13.78%) in CN2. Treatment CN3 showed the highest relative abundance of Planctomycetota (14.61%). The highest relative abundance of Acidobacteriota was observed in the CK treatment.

Comparing different fertilization practices, treatments combining organic and chemical fertilizers exhibited an increasing trend in the relative abundances of Proteobacteria, Planctomycetota, and Bacteroidota. Their relative abundances were higher than those in the single-fertilizer treatments (NC0 and CN0) and CK. Conversely, the relative abundance of Acidobacteriota was highest, intermediate, and lowest in CK, the single-fertilizer treatments, and the treatments combining organic and chemical fertilizers, respectively.

### 2.5. Soil Microbial Community Beta Diversity Under Reduced Nitrogen Combined with Organic Fertilizer Application

**Figure 3:**
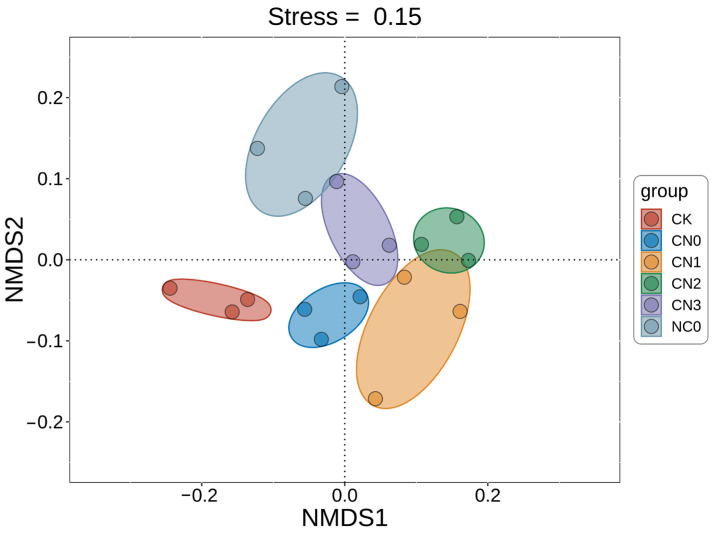
NMDS analysis of bacterial community structure in maize rhizosphere soil across treatments. NC0: 180 kg N/ha; CN0: 3000 kg OF/ha; CN1: OF (3000 kg/ha) + Reduced N (90 kg N/ha, 50% reduction); CN2: OF (3000 kg/ha) + Reduced N (135 kg N/ha, 35% reduction); CN3: OF (3000 kg/ha) + Conventional N (180 kg N/ha, 0% reduction).

A beta diversity analysis of the soil bacterial communities across treatments was performed using non-metric multidimensional scaling (NMDS). As depicted in Figure 3, the control group (CK) and the zero-nitrogen treatment (CN0) were distinctly separated from all other treatment groups. While a slight overlap was observed between the conventional nitrogen fertilization treatment (NC0) and CN3, both were clearly separated from the remaining treatments. Conversely, the treatment groups combining organic and chemical fertilizers (CN1, CN2, and CN3) exhibited considerable overlap and clustered closely together.

### 2.6. The Relationship Between Soil Physicochemical Properties, Yield Components, and Microbial Diversity

**Figure 4:**
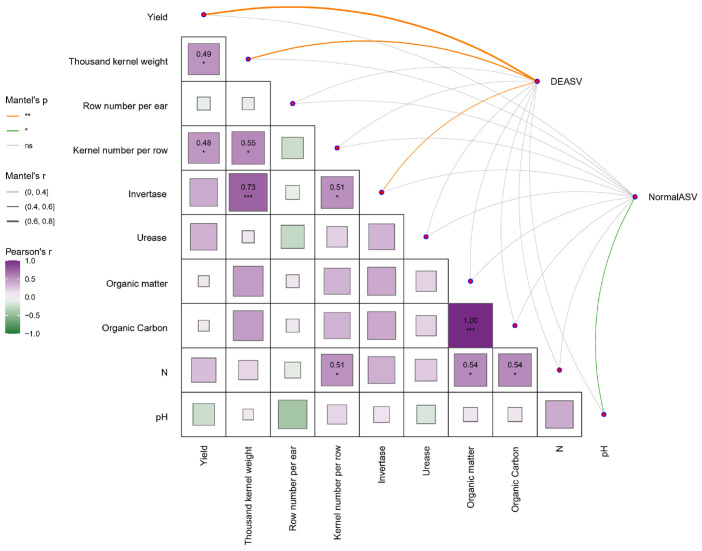
Mantel test between soil physicochemical properties/yield factors and microbial diversity. Edge width corresponds to the absolute value of the correlation coefficient determined by the linear mixed-effects models. Colors indicate correlation types. Lines of different colors represent significant and non-significant correlations determined based on the mantel tests. Pairwise comparisons of environmental factors are shown in the triangle, with a color gradient denoting Pearson’s correlation coefficient. The *p* values were adjusted by the false discovery rate; *** *p* < 0.001, ** *p* <  0.01, * *p* <  0.05.

Correlation analysis was performed between maize yield components, soil nutrient/environmental factors, and the microbial community. We found correlations between DEASV/NormalASV and both maize yield and soil environmental factors. As shown in Figure 4, DEASV exhibited a significant positive correlation with maize yield and soil invertase activity. Conversely, NormalASV showed a significant negative correlation with soil pH. Regarding yield components, both kernels per row and 1000-kernel weight showed significant positive correlations with yield. Soil invertase activity exhibited an extremely significant positive correlation with 1000-kernel weight and a significant positive correlation with kernels per row. The soil AN content showed significant positive correlations with the kernels per row, SOM, and SOC content. To further elucidate the relationships between microbial community composition, soil environment, and maize yield, a correlation analysis was conducted at the phylum level between the relative abundance of microorganisms and soil environmental factors/yield components.

**Figure 5:**
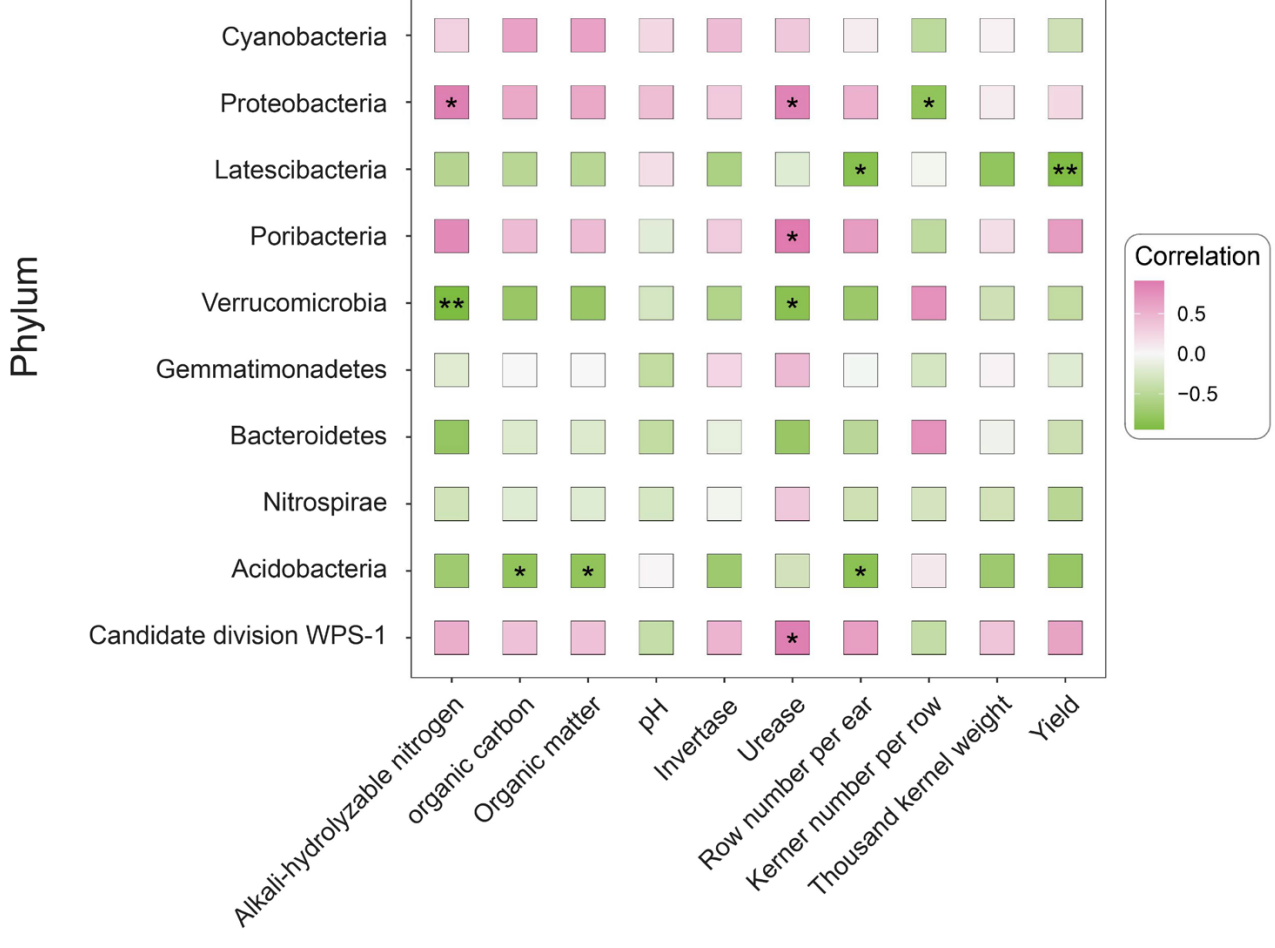
Correlation analysis between soil physicochemical properties/yield factors and microbial species. The *p* values were adjusted by the false discovery rate; ** *p* <  0.01, * *p* <  0.05. The color of the square is pink, indicating a positive correlation between the two factors. Green indicates a negative correlation between the two factors, with color filling indicating the strength of the correlation.

As depicted in Figure 5, among the top ten most abundant microbial phyla, different phyla exerted differential impacts on the soil environment and crop yield, primarily influencing soil organic matter content, alkali-hydrolyzable nitrogen content, urease activity, and maize yield. The key correlations included the following: Proteobacteria exhibited a positive correlation with soil AN content and urease activity and a negative correlation with rows per ear. Patescibacteria exhibited a negative correlation with kernels per row and an extremely significant negative correlation with yield. Poribacteria exhibited a positive correlation with soil urease activity. Verrucomicrobiota exhibited a negative correlation with urease activity and an extremely significant negative correlation with the soil AN content. Acidobacteriota exhibited a negative correlation with the soil SOM, SOC, and kernels per row. Candidate division WPS-1 exhibited a positive correlation with soil urease activity.

## 3. Discussion

### 3.1. Effects of Reduced Nitrogen Combined with Organic Fertilizer Application on Maize Yield and Yield Components

In this study (Table 1), the treatment combining a conventional nitrogen fertilizer with an organic fertilizer (CN1) achieved the highest grain yield, significantly outperforming both the no-fertilizer control (CK) and the sole organic fertilizer treatment (CN0). These findings not only validate the yield advantage of the chemical–organic fertilizer co-application model but also reveal the unique physiological effect of organic fertilizer in promoting grain filling. This aligns closely with the conclusions of Nie [34] in the wheat–maize rotation system of the Huang–Huai–Hai Plain, demonstrating that conventional chemical fertilizer combined with organic fertilizer significantly increases crop yield compared to sole chemical or organic fertilizer application, favoring sustainable production in wheat–maize systems. Studies by Wang and Mao [16,35] reported dramatic yield increases (7-fold and 14.8-fold higher than the control, respectively) under combined organic and chemical fertilization. Although the magnitude exceeded that observed here, they similarly corroborate the immense yield potential of the co-application model. The core mechanism underlying this yield enhancement lies in the synergistic effect of chemical and organic fertilizers, which significantly improves fertilizer use efficiency, reduces nutrient loss (especially nitrogen) or fixation, and thereby ensures more sufficient and balanced nutrient supply during critical growth stages, ultimately translating into higher grain yields.

This study (Figure 4) demonstrated that 1000-kernel weight and grains per row were significantly correlated with yield, indicating that increased fertilizer application influences yield primarily through its effects on 1000-kernel weight and grains per row. Among the treatments, CN0 showed the greatest increase in 1000-kernel weight relative to CK, while CN1 exhibited the maximum increases in grains per row and yield compared to CK. These findings align with previous research [36] on reduced nitrogen application rates with organic amendments. The author proposed that organic fertilizers help delay vegetative organ senescence, enhancing photosynthetic accumulation throughout the growth period while increasing dry matter partitioning to grains. In this study (Figure 4), invertase activity showed a significant correlation with 1000-kernel weight, suggesting that enhanced sucrose hydrolysis by invertase may increase the photosynthate supply to developing grains.

### 3.2. Effects of Reduced Nitrogen Combined with Organic Fertilizer on Soil Nutrient Content and Enzyme Activity

This short-term fertilization experiment revealed the following differential impacts of combined organic–inorganic fertilization on summer maize soil in southern Shanxi: (1) Compared to the no-fertilizer control (CK), both sole chemical fertilizer (NC0) and combined application treatments significantly increased the SOM and AN content. However, the combined treatments did not yield significant additional gains over sole chemical fertilizer (NC0). (2) In contrast, the combined application treatments consistently and significantly enhanced soil urease and invertase activities, far exceeding the levels in sole chemical fertilizer (NC0) and CK. These results highlight that, over the short term, key soil enzyme activities exhibit greater sensitivity to organic inputs than fundamental nutrient pool indicators. SOM and AN are core indicators for assessing inherent soil fertility, directly influencing soil nutrient supply capacity and crop productivity. The observed significant increases in SOM and AN in fertilized treatments (NC0 and combined) compared to CK directly confirm that fertilizer inputs (whether chemical or organic) are key drivers for maintaining and enhancing the fundamental soil nutrient pool [37,38]. However, within the short experimental duration, combined organic fertilizer application did not generate significant additional gains in the SOM and AN content over sole chemical fertilizer (NC0). This contrasts with results from many long-term field experiments [39,40]. This discrepancy underscores the long-term and cumulative nature of organic amendments in rebuilding the recalcitrant soil C and N pools [41]. Under short-term application, the readily decomposable components of organic fertilizers can be rapidly mineralized to release nitrogen, which is primarily utilized by plants. Consequently, their contribution to soil nutrient pools remains undetectable in the short term. The significant contribution of these components to the stable soil organic matter (SOM) pool only becomes apparent after multi-year accumulation through humification processes.

The combined treatments significantly boosted the activities of these two key extracellular enzymes, surpassing NC0 and CK. Soil enzymes are core biological catalysts driving organic matter decomposition and nutrient transformation, regulated by substrate availability, microbial activity, and the soil microenvironment [42]. Therefore, the enzyme activity results indicate that, even without significantly boosting the fundamental SOM pool capacity in the short term, the combined organic fertilizer application rapidly activated soil microbial function, thereby strengthening the soil’s nutrient transformation and turnover capacity.

### 3.3. Effects of Reduced Nitrogen Combined with Organic Fertilizer on Soil Bacterial Community

As a core biological indicator of ecosystem functional stability, soil microbial community diversity dynamically reflects the interplay between soil environmental factors and anthropogenic interventions [43,44]. Studies show that soil physicochemical properties significantly regulate microbial community composition, functional gene distribution, and interspecies interaction networks by shaping fundamental niche conditions [35,45]. The results of this study showed that compared to the control (CK) and sole fertilization treatments, combined organic fertilizer application significantly increased the Chao1 and ACE indices of the soil microbial community, while the Shannon indices showed no significant increase (Figure 1). This indicates that fertilization increases the richness of the soil microbial community but does not affect its evenness. This aligns with the findings of Ma et al. [46], supporting the potential of fertilization to create new niche space in low-fertility soils. However, long-term field experiments conducted in the black soil region [47] demonstrated that both mineral fertilizer application and the combined application of organic and inorganic fertilizers significantly reduced soil bacterial species richness. The farmland in the present study employed a wheat–maize rotation system, whereas the long-term experiment in the black soil region utilized a wheat–maize–soybean rotation system. The inclusion of soybean alters nitrogen cycling patterns through biological nitrogen fixation, whereas nitrogen in the wheat–maize system of this study is primarily supplied via exogenous fertilizer application. Concurrently, the black soil region exhibits higher inherent soil fertility; consequently, exogenous fertilizer inputs may impose stress on certain microbial groups, leading to a decline in microorganisms sensitive to high-nutrient environments and thereby reducing bacterial species richness. In contrast, the experimental site in this study had lower initial fertility, whereby exogenous nutrient inputs are more likely to create new ecological spaces. We speculate that the observed stability arises because the fertilization treatments while increasing the absolute abundance of various bacterial taxa in concert, did not alter the relative abundance patterns among taxonomic units, thereby maintaining the overall structural equilibrium of the community. The stability in evenness indices suggests that while fertilization increased the absolute abundance of various bacterial groups, the relative proportional structure among dominant taxa maintained a dynamic equilibrium. This aligns with the functional redundancy hypothesis, where microbial communities buffer disturbances by maintaining the stability of key functional groups, ensuring the continuity of core ecosystem processes [48].

The NMDS analysis revealed that different fertilization treatments significantly altered the soil microbial community structure (Figure 3). The CK and sole organic fertilizer (CN0) treatments were distinctly separated from other treatments. This indicates that fertilization acted as an environmental driver, promoting the restructuring of the microbial community. Nutrient input drove community reconstruction, while the nutrient source further shaped community specificity. The sole organic fertilizer treatment (CN0) inputted structurally complex organic matter and thus likely selectively enriched specialized microbial taxa possessing corresponding decomposition capabilities, forming a community configuration distinct from treatments reliant on readily available chemical nutrients (NC0 and combined applications).

NormalASV was more influenced by pH, potentially representing a widely adapted community baseline (Figure 4). This is consistent with previous studies identifying soil pH as a key factor governing microbial community shifts [49]. Conversely, DEASV points to a functionally active microbial assemblage sensitive to fertilization practices. In this study, DEASV showed strong correlations with yield, 1000-kernel weight, and invertase activity, indicating its reliance on responses linked to soil enzyme activity, particularly carbon metabolism mediated by invertase activity, indirectly regulating crop yield and kernel weight. This suggests that these functional microbes may enhance soil carbon turnover efficiency, optimizing carbon assimilate supply to the crop, thereby directly driving productivity gains.

The dominant bacterial phyla across all treatments were Proteobacteria, Acidobacteriota, Planctomycetota, and Bacteroidota (Figure 2), consistent with previous reports [50,51]. Combined organic–inorganic fertilization significantly altered soil bacterial community structure. Specifically, the relative abundance of Proteobacteria in treatment CN2 increased by 5.73% and 3.42% compared to CK and sole chemical fertilizer (NC0), respectively. As key functional groups in carbon and nitrogen cycling, the increased abundance of Proteobacteria species may relate to their rapid response to soluble organic carbon in root exudates. Acidobacteriota, classified as oligotrophic bacteria adapted to low-nutrient environments, showed enrichment in sole fertilization treatments in this study, reflecting a shift from an oligotrophic soil environment towards a mesotrophic state. The proposed niche differentiation mechanism is as follows: exogenous nutrient inputs improve crop nutritional status, promoting root biomass growth and changes in exudate composition; this alteration in the rhizosphere microenvironment intensifies plant–microbe interactions. Soil urease activity showed a positive correlation with Proteobacteria, Poribacteria, and candidate division WPS-1 and a negative correlation with Verrucomicrobiota (Figure 5). This correlation reflects the distinct roles of different microbial groups in the soil nitrogen cycle. Proteobacteria, the dominant phylum in soils, exhibited significant positive correlations with urease activity and available nitrogen (AN) in this study (Figure 5). Organic fertilizer application increased Proteobacteria abundance by providing abundant nutrient and energy substrates [52]. Concurrently, urea application stimulated enhanced urease expression [53], particularly under alkaline conditions (pH 7–9) where elevated ammonium concentrations promote urease production [54]. We therefore speculate that under combined organic–inorganic fertilization regimes, these inputs jointly stimulate both Proteobacteria enrichment and urease expression. This study found correlations between urease activity and Poribacteria, candidate division WPS-1, and Verrucomicrobiota that differ from some of the existing literature reports. Existing studies suggest that Poribacteria participates in carbohydrate metabolism, primarily degrading glycosaminoglycan chains of proteoglycans [55]. Its positive correlation with urease activity here might stem from degrading extracellular polysaccharides of urease-producing bacteria, indirectly enhancing urease activity. Candidate division WPS-1, primarily anaerobic, is mainly associated with phosphorus cycling [56], while Verrucomicrobiota abundance is often regulated by nematode symbiosis [57]. The observed direct functional links to urease activity in this study may arise from the non-canonical ecological roles that these microbial groups adopt in specific soil environments.

## 4. Materials and Methods

### 4.1. Experimental Site

The experiment was conducted in 2023 at the Experimental Base of the Cotton Research Institute, Shanxi Agricultural University, Yuncheng City, Shanxi Province (35°06′33″ N, 110°55′57″ E). The site was a wheat–maize rotation field under a double-cropping system. Annual precipitation (mean: 500 mm) primarily occurs from June to September, with a mean annual temperature and frost-free period of 13.5 °C and 210 days, respectively. The experimental site featured Haplic Luvisol (FAO classification), locally termed cinnamon soil. The initial properties of the tillage layer (0–20 cm depth) were characterized as follows:

Soil organic matter (SOM): 10.49 g kg^−1^;
Total nitrogen (TN): 0.867 g kg^−1^;
Alkali-hydrolyzable nitrogen (AN): 30.70 mg kg^−1^;
Available phosphorus (AP): 9.31 mg kg^−1^;
Available potassium (AK): 156.5 mg kg^−1^;
pH (soil: water = 1:2.5): 8.1.

### 4.2. Experimental Design

The maize cultivar ‘Lenong 87’ was used. A randomized complete block design with 6 treatments, each with 3 replicates, was implemented (a total of 18 plots; plot area = 15 m^2^ (6 m × 2.5 m)):

CK: No fertilizer;
NC0: Conventional nitrogen (N) application (180 kg N/ha);
CN0: Conventional organic fertilizer (OF) application (3000 kg OF/ha);
CN1: OF (3000 kg/ha) + Reduced N (90 kg N/ha, 50% reduction);
CN2: OF (3000 kg/ha) + Reduced N (135 kg N/ha, 35% reduction);
CN3: OF (3000 kg/ha) + Conventional N (180 kg N/ha, 0% reduction).

The nitrogen fertilizer was applied as urea (46% N). A commercial organic fertilizer (total N + P_2_O_5_ + K_2_O ≥ 5%; organic matter ≥ 45%; viable bacteria count ≥ 5 × 10^8^ CFU/g) was used. All treatments received equal amounts of phosphorus and potassium fertilizer (monopotassium phosphate; KH_2_PO_4_; 99% purity). All fertilizers were applied basally before sowing. Maize was sown on 16 June 2023, and harvested on 24 October 2023.

### 4.3. Sample Collection

At the mid-grain filling stage (September 14), rhizosphere soil samples were collected from the 0–20 cm plow layer of each plot. Rhizosphere soil was collected by gently shaking the roots to detach loosely adhered soil, followed by sieving (2 mm) to remove root debris. Following root removal, all samples were sieved (<2 mm) and divided into two subsamples. The homogenized samples were divided as follows:

Subset 1: Stored in sterile PVC tubes at −80 °C for microbiological/enzymatic assays.
Subset 2: Air-dried, sieved (2 mm), and stored for physicochemical analysis [58].

### 4.4. Measurements

Soil Microbiome Analysis:

Total DNA was extracted using the PowerSoil^®^ DNA Isolation Kit (MO BIO, D3142-03, MO BIO Laboratories, Inc., Carlsbad, CA, USA). The V3–V4 region of bacterial 16S rRNA was amplified with primers 338F/806R [59] under standardized PCR conditions (95 °C/3 min; 30 cycles of 95 °C/30 s, 55 °C/30 s, 72 °C/45 s; final extension: 72 °C/10 min). Amplicons were validated by 2% agarose gel electrophoresis, purified, and sequenced on an Illumina MiSeq platform (Hualianke Biotech, Wuhan, China). Raw reads were processed via FLASH (v1.2.11) [60], clustered into OTUs at 97% similarity (UPARSE v7.0.1090) [61], and taxonomically classified against the SILVA database (RDP classifier, v2.11; confidence threshold: 0.7) [62].

Soil physicochemical properties [63,64,65]:

SOM: Potassium dichromate oxidation-external heating method.
AN: Alkaline diffusion method.
pH: Glass electrode (PHS-3E, INESA) in 1:5 soil–water suspension.
Urease activity: Indophenol blue method (μg NH_4_^+^-N g^−1^ h^−1^).
Invertase activity: DNS method (mg glucose g^−1^ 24 h^−1^).

Yield and yield components:

The harvest density was determined pre-harvest. At maturity, the ears were harvested from three sampling points per plot. Ear weight was recorded. The kernels were threshed, and the grain moisture was measured using a grain moisture meter. The grain yield was calculated at a 14% moisture content. For yield components, 10–15 representative ears were selected to measure the following: Rows per ear/Kernels per row/1000-kernel weight.

### 4.5. Data Processing and Analysis

Primary data organization was performed in Microsoft Excel 2021. Statistical analyses included one-way ANOVA with Tukey’s HSD test (*p* < 0.05) for treatment effects (SAS 9.4); Pearson correlation analysis between soil microbiological, enzymatic, and yield parameters; a principal component analysis (PCA) of microbial community structure; and the visualization of results using ggplot2 package in R (v4.3.1).

## 5. Conclusions

Reducing nitrogen fertilization while incorporating organic amendments represents a transformative strategy for sustainable maize production. This approach synergistically enhances yield while fundamentally improving soil biological fertility. The optimal treatment (3000 kg/ha organic fertilizer + 90 kg/ha nitrogen) achieved a high grain yield (9764.87 kg/ha) with reduced chemical inputs. Further increasing the nitrogen application by 45 kg/ha maintained comparable yields while delivering higher enzyme activities and microbial abundance, positioning this strategy as suitable for soil remediation. Crucially, organic inputs significantly elevated key soil enzyme activities (23.07% increase in urease activity), restructured microbial communities, and enriched functional taxa that were positively correlated with N-cycling enzymes (Proteobacteria, Poribacteria, and candidate_division_WPS-1) while suppressing negatively associated phyla (Verrucomicrobia). These findings demonstrate that nitrogen reduction through organic amendments optimizes soil microbial community structure, thereby enhancing crop productivity through improved nutrient cycling and soil health. This provides scientific foundations for sustainable agricultural ecosystems.

## Figures and Tables

**Table 1:** Maize yield and yield components under different treatments.

Treatments	Yield (kg/ha)	1000-Kernel Weight (g)	Rows per Ear	Grains per Row	Number of Ears per Hectare
CK	6649.30 b	203.17 c	16.60 a	29.1 b	62,500 ab
NC0	9031.70 a	264.33 ab	17.40 a	31.2 ab	65,741 ab
CN0	7455.41 b	280.83 a	16.20 a	32.4 ab	61,111 b
CN1	9764.87 a	263.83 ab	16.20 a	33.70 a	70,730 ab
CN2	9474.75 a	268.17 ab	16.60 a	33.30 a	69,444 ab
CN3	9303.28 a	253.17 b	16.60 a	32.20 ab	72,222 a

Note: Different lowercase letters following values within a row indicate significant differences among treatments at *p* < 0.05. Same for the following tables.

**Table 2:** Soil nutrients, enzyme activities, and pH under different treatments.

Treatment	SOM g/kg	AN mg/kg	Urease U/g	Invertase U/g	pH
CK	10.99 b	47.87 a	4287.10 ab	53.61 c	8.83 ab
NC0	11.73 ab	52.50 a	3752.9 b	61.59 bc	8.76 b
CN0	13.55 ab	54.27 a	4367.8 a	70.84 a	8.90 a
CN1	13.33 ab	64.77 a	4739.7 a	64.74 ab	8.90 a
CN2	13.99 a	58.33 a	4878.6 a	69.70 ab	8.74 a
CN3	11.25 ab	53.83 a	4833.6 a	61.92 b	8.74 a

Note: Different lowercase letters following values within a row indicate significant differences among treatments at *p* < 0.05. SOM: soil organic matter. AN: alkali-hydrolyzable nitrogen.

## Data Availability

Data are contained within the article.
